# Statin use and risk of depression: a Swedish national cohort study

**DOI:** 10.1186/s12888-014-0348-y

**Published:** 2014-12-04

**Authors:** Cassie Redlich, Michael Berk, Lana J Williams, Jan Sundquist, Kristina Sundquist, Xinjun Li

**Affiliations:** Center for Primary Health Care Research, Lund University, Lund, Sweden; IMPACT Strategic Research Centre, School of Medicine, Deakin University, Locked Bag 20000, Geelong, 3220 Australia; Department of Psychiatry, The University of Melbourne, Parkville, Australia; Stanford Prevention Research Center, Stanford University School of Medicine, Stanford, California USA

**Keywords:** Statins, Atorvastatin, Simvastatin, Depression, Aetiology, Inflammation, Oxidative stress

## Abstract

**Background:**

Statin medications, used to prevent heart disease by reducing cholesterol, also reduce inflammation and protect against oxidative damage. As inflammation and oxidative stress occur in depression, there is interest in their potential to reduce depression risk. We investigated whether use of statin medications was associated with a change in the risk of developing depression in a very large Swedish national cohort (n = 4,607,990).

**Methods:**

National register data for adults ≥40yr was analyzed to obtain information about depression diagnoses and prescriptions of statin medications between 2006 and 2008. Associations were tested using logistic regression.

**Results:**

Use of any statin was shown to reduce the odds of depression by 8% compared to individuals not using statin medications (OR = 0.92, 95% CI, 0.89-0.96; p < 0.001). Simvastatin had a protective effect (OR = 0.93, 95% CI, 0.89-0.97; p = 0.001), whereas atorvastatin was associated with increased risk of depression (OR = 1.11, 95% CI, 1.01-1.22; p = 0.032). There was a stepwise decrease in odds ratio with increasing age (OR ≥ 40 years = 0.95, OR ≥ 50 years = 0.91, OR ≥ 60 years = 0.85, OR ≥ 70 years = 0.81).

**Conclusions:**

The use of any statin was associated with a reduction in risk of depression in individuals over the age of 40. Clarification of the strength of these protective effects, the clinical relevance of these effects and determination of which statins are most effective is needed.

## Background

Depression is an episodic condition with recurrence common across the lifespan [1-3], and a typical relapse rate of between 50-70% depending on number of previous episodes [1]. An inflammatory model of depression has been put forward in recent years, highlighting the role of inflammatory, oxidative and nitrosative stress (IO&NS) pathways in the development of depression and conceptualizing depression within the spectrum of neuroprogressive disorders [4,5]. Previous evidence supporting this model has shown pro-inflammatory cytokines to be increased in patients with depression [6], disease states characterized by inflammation are often accompanied by depressive illness [7,8], and patients exposed to pro-inflammatory cytokines often report subsequent development of depression [9]. Inflammatory responses are known to be accompanied by stimulation of IO&NS pathways and the production of free radicals [4], which can lead to oxidation and cell damage over time if not regulated. There is parallel evidence that markers of oxidative damage appear to be increased in patients diagnosed with depression [4], leading to the hypothesis that agents targeting inflammation and IO&NS pathways may be effective in both treating and reducing the risk of depression.

Statins, or 3-hydroxy-3-methylglutaryl coenzyme A reductase inhibitors, are commonly prescribed lipid-lowering drugs used primarily in the treatment of high cholesterol and cardio-vascular disease [10]. Evidence suggests that statins may also have a neuroprotective quality [10], with studies finding that statin use may be associated with a reduction in the incidence of dementia [11], Alzheimer’s disease [12] and Parkinson’s disease [13]. Statins have also been linked to possible neurocognitive effects [14]. It has been hypothesized that the effects may be due to action at the cellular level, or may be as a result of their ability to reduce oxidative stress [15,16] and to modulate inflammatory responses [9,10].

Given the anti-inflammatory and anti-oxidative properties of statins, recent authors have hypothesized that treatment with statins may have a protective effect on the development of depression. This hypothesis is in contrast to previous concerns that lowered cholesterol levels could have a negative impact on mood and behaviour, with a number of early studies suggesting that low levels of serum cholesterol (both naturally low and medically lowered) were associated with lowered mood and increased anger, hostility, aggression and possible suicidal ideation [17]. Later studies investigating the effects of statins specifically on mood have reported mixed findings, with some suggesting an increased risk of depression [18] and decrease in positive affect [19], while others have found either no association between statin use and mood disturbances [20-23], or that stains in fact may protect against the risk of depression [9,17,24-27]. A recent meta-analysis has supported the latter conclusion [5]. Clarification of this association appears warranted, as confirmation that statin use is effective in reducing the risk of subsequent depression could firstly lend proof of principle support to the inflammatory model of depression and may additionally lead to the development of new treatment approaches. This data is additionally important for clarifying the risk-benefit profile of statins, given their status as one of the most widely prescribed agents in medicine.

### Aims of the study

The objective of the current study is to investigate the relationship between use of statin medications and development of depression in a large Swedish population cohort. The effect of statin medications as a group as well as the effect of individual statins will be examined.

## Methods

### Data sources

This study is based on anonymous data from census, pharmacy, inpatient and outpatient registers for the entire Swedish population aged 40 years and over. Individual-level demographic information was obtained using national census data from 2005. Information regarding prescription of statin medications was obtained from the National Pharmacy Register, which is maintained by the Swedish National Board of Health and Welfare. This register contains a record of each medication prescribed by a health care provider and dispensed by any outpatient or inpatient pharmacy in Sweden from July 1, 2005. The medication data are classified according to the Anatomical Therapeutic Chemical (ATC) System developed by the World Health Organization Collaborating Centre for Drug Statistics Methodology. Data was obtained for medications classified as lipid modifying agents (code C10A), sub-classified as HMGCoA reductase inhibitors (statins, codes C10AA01 - 08).

Information regarding hospitalization for chronic obstructive pulmonary disease (COPD), alcohol dependence and coronary heart disease (CHD) was obtained from the Swedish National Inpatient Register (IPR), also maintained by the Swedish National Board of Health and Welfare. The IPR contains diagnostic and administrative information regarding all somatic and psychiatric hospital discharges in Sweden from 1987 and 1973 respectively. Diagnoses in the IPR are classified according to the International Classification of Disease (ICD) system developed by the World Health Organization.

### Study population

The study population consisted of all men and women over the age of 40 identified as living in Sweden as of January 1, 2006. We excluded 11695 individuals who were identified as having had a diagnosis of depression that preceded a prescription of statin medication during the study period (0.25%). A total of 4,607,990 individuals remained for inclusion in the analysis. The study cohort was followed from January 1, 2006 through December 31, 2008.

### Outcome variable

The outcome of interest was a diagnosis of depression (ICD-10 codes F30 - F39) between January 1, 2006 and December 31, 2008, by any inpatient or outpatient health care provider in Sweden and by initiation of treatment as indicated by medication data (ATC codes N06A) during the study period. Only the first diagnosis for an individual during the study period was included.

### Independent variables

The association of interest was prescription of a statin medication dispensed at any outpatient or inpatient pharmacy in Sweden during the study period. This variable was evaluated for prescription of any statin, and by individual statins (simvastatin, pravastatin, fluvastatin, atorvastatin, rosuvastatin lovastatin and pitavastatin). Prescription of a statin medication was taken as a proxy for statin use during the study period, with the assumption made that individuals receiving a prescription would proceed to take the medication as prescribed.

Other independent variables included gender, age [divided into 10-year categories (40–49 years, 50–59 years, 60–69 years, and over 70 years)], immigration status (born in Sweden, or born outside of Sweden) and marital status (grouped as married/cohabitating or unmarried/widowed/divorced/single). Education was obtained and grouped as completion of compulsory school or less (≤9 years), practical high school or some theoretical high school (10-11 years), or theoretical high school and or college (≥12 years). Family income was obtained from the Total Population Register provided by Statistics Sweden. This information was used to determine the distribution of family incomes in Sweden, categorised into quartiles (low, middle low, middle high, and high income). Region of residence was categorised as large cities (with a population over 200 000, i.e. Stockholm, Gothenburg and Malmö), Southern Sweden, and Northern Sweden. Previous hospitalisation for chronic obstructive pulmonary disease (COPD, ICD-10 code J40-49), which was suspected to be an important prognostic factor for depression as a proxy for smoking, was dichotomized into yes or no. Previous hospitalisation for alcoholism and related liver disease (ICD-10 codes F10 and K70) was dichotomized into yes or no. Previous hospitalisation for coronary heart disease (CHD, ICD-10 codes I20 - I25) was also dichotomized into yes or no.

### Statistical analysis

The incidence rate for depression over the study period was calculated for each variable separately by dividing the number of diagnoses by the total number of individuals in each category. The calculations were made stratifying by age, and the results then multiplied by the relative proportions within each age group and added together to obtain the age-standardized incidence rates. As the outcome of interest was a binary categorical variable, logistic regression was used to estimate odds ratios (ORs) and 95% confidence intervals (95% CI) to represent the strength of association between use of a statin medication (any statin and by individual statins, vs. no prescription of statin medication) and subsequent diagnosis of depression during the study period. Analyses were conducted unadjusted, age-adjusted, and then were adjusted for other independent variables in order to account for confounding (gender, marital status, immigration status, education, family income, region of residence, and previous hospitalization for COPD, alcohol dependence and related liver disease, and CHD). Adjusted odds ratios for the association between use of statin medications and subsequent diagnosis of depression were then compared across age groups (≥40 years, ≥50 years, ≥60 years, ≥70 years). It was decided a priori that significance would be accepted at p < 0.05. All analyses were carried out using Stata statistical software, version 11.2 [28].

### Ethical considerations

Data from the registers utilised in this study were linked using an anonymous, serial number version of the personal identification number provided to all Swedish residents, ensuring confidentiality of the data. This study was approved by the Ethics Committee of Lund University, Sweden.

## Results

All individuals 40 years of age and older living in Sweden at the start of the study period, totaling 4,607,990 individuals, were included in the analysis. The age range was from 40 to 111 years of age, with a mean age of 60. Individual characteristics of the cohort, including number of depression diagnoses by independent variable and age-adjusted incidence rates (p/1000 population) during the study period are presented in Table 1. The age distribution of the cohort was fairly even across the four age groups, with slightly more in the 40-49 year (26.9%), and less in the 60-69 year old category (22.4%). Most individuals (46%) had completed 12 years of schooling or more. Those born outside of Sweden accounted for almost 14% of the total number, and approximately half the cohort (49%) was residing in large cities at the start of the study period.

**Table 1:** **Individual characteristics, number of depression cases by independent variable, and age-standardized rates (p/1000 population) for diagnosis of depression between 2006-2008 (** ***n*** **= 4,607,990)**

	**Population**	**% of total population**	**Number of cases**	**Rate**	**95% CI**
**Age**					
40-49	1,238,099	26.9	5,607	4.53	4.38-4.62
50-59	1,190,481	25.8	5,048	4.24	4.08-4.32
60-69	1,033,816	22.4	3,886	3.76	3.68-3.92
70+	1,145,594	24.9	5,751	5.02	4.87-5.13
**Gender**					
Male	2,220,494	48.19	8,317	3.75	3.67-3.83
Female	2,387,496	51.81	11,975	4.99	4.90-5.08
**Civil status**					
Married/cohabitating	2,498,864	54.23	7,456	3.05	2.98-3.12
Unmarried/widowed/divorced/single	2,109,126	45.77	12,836	6.09	5.98-6.20
**Immigration**					
Born in Sweden	3,968,191	86.12	17,298	4.36	4.30-4.42
Born elsewhere	639,799	13.88	2,994	4.63	4.46-4.80
**Education**					
Compulsory school or less (≤ 9yrs)	1,244,872	27.02	6,384	5.03	4.91-5.15
Practical high school or some theoretical high school (10-11yrs)	1,243,067	26.98	5,841	4.76	4.64-4.88
Theoretical high school and/or college (≥ 12yrs)	2,120,051	46.01	8,067	3.93	3.84-4.02
**Income**					
Low income	1,018,845	22.11	5,161	5.08	4.94-5.22
Middle low income	1,219,372	26.46	7,421	6.15	6.01-6.29
Middle high income	1,151,642	24.99	4,589	3.99	3.87-4.11
High income	1,218,131	26.44	3,121	2.79	2.69-2.89
**Hospitalisation for COPD**					
No	4,581,872	99.43	19,908	4.35	4.29-4.41
Yes	26,118	0.57	384	19.86	17.87-21.85
**Hospitalisation for alcohol dependence**					
No	4,583,249	99.46	18,856	4.12	4.06-4.18
Yes	24,741	0.54	1,436	54.34	51.53-57.15
**Hospitalisation for CHD**					
No	4,496,433	97.6	19,599	4.37	4.31-4.43
Yes	111,557	2.4	693	7.02	6.50-7.54
**Region**					
Large cities	2,259,664	49.04	10,452	4.64	4.55-4.73
Southern	1,579,534	34.28	6,617	4.19	4.09-4.29
Northern	768,792	16.68	3,223	4.2	4.05-4.35
**Statin prescriptions**					
Any statin	804,832	17.47	3,313	4.59	4.43-4.75
Simvastatin	665,932	14.45	2,707	4.45	4.28-4.62
Pravastatin	27,244	0.59	103	5.21	4.20-6.22
Fluvastatin	5,932	0.13	24	5.07	3.04-7.10
Atorvastatin	95,231	2.07	429	5.49	4.97-6.01
Rosuvastatin	10,493	0.23	50	5.11	3.69-6.53

IR = age-standardized incidence rate; CI = confidence interval.

COPD = chronic obstructive pulmonary disease.

CHD = coronary heart disease.

During the study period, 804,832 prescriptions for statin medication were dispensed. Of these prescriptions, approximately 70% were prescribed to individuals aged 60 years and over, with the proportion of total prescriptions increasing by increasing age group. No prescriptions for lovastatin or pitavastatin were recorded during the study period, therefore they were excluded from the analysis. Of the remaining statins, simvastatin was by far the most widely prescribed, accounting for 83% of total statin prescriptions (*n* = 665,932).

Being a woman, single, having 9 years or less of education, and having a history of hospitalization for COPD, alcoholism or ischemic heart disease were associated with relatively higher rates of depression within the cohort (Table 1).

Table 2 presents the unadjusted and adjusted ORs from for the association between statin prescription and subsequent diagnosis of depression. Use of any statin medication reduced the odds of subsequently developing depression by approximately 8% compared to individuals who had not used statin medications (OR = 0.92, 95% CI, 0.89-0.96; p < 0.001). After adjusting for potential confounding variables, this result was slightly attenuated (OR = 0.95, 95% CI, 0.91-0.99; p = 0.016). Use of simvastatin similarly was associated with a reduction in the odds of developing depression (OR = 0.93, 95% CI, 0.89-0.97; p = 0.001). Conversely, prescription of atorvastatin was associated with an 11% increase in the odds of developing depression (OR = 1.11, 95% CI, 1.01-1.22; p = 0.032). The relationship between depression and other individual statin medications was not found to be significant.

**Table 2:** **Unadjusted and adjusted ORs from logistic regression for association between statin prescription and subsequent diagnosis of depression between 2006-2008 (** ***n*** **= 4,607,990)**

	**Unadjusted OR**	**95% CI**	**p-value**	**Age-adjusted OR**	**95% CI**	**p-value**	**Adjusted OR** ^**a**^	**95% CI**	**p-value**
**Statin prescriptions**									
Any statin	0.92	0.89-0.96	<0.001	0.92	0.88-0.95	<0.001	0.95	0.91-0.99	0.016
Simvastatin	0.91	0.87-0.95	<0.001	0.91	0.87-0.94	<0.001	0.93	0.89-0.97	0.001
Pravastatin	0.86	0.71-1.04	0.120	0.85	0.70-1.03	0.090	0.90	0.74-1.09	0.268
Fluvastatin	0.92	0.61-1.37	0.677	0.91	0.61-1.36	0.643	0.98	0.65-1.46	0.914
Atorvastatin	1.02	0.93-1.13	0.632	1.03	0.94-0.97	0.507	1.11	1.01-1.22	0.032
Rosuvastatin	1.08	0.82-1.43	0.578	1.12	0.84-1.47	0.443	1.21	0.92-1.60	0.177

CI = confidence interval; OR = odds ratio.

^**a**^The model includes prescription of statin medication (any statin, and by individual statins) and the following variables as covariates: gender, age (40-49, 50-59, 60-69, 70+), marital status, education (three levels), family income (quartiles), hospitalisation for cardio-pulmonary disease, hospitalisation for alcoholism, hospitalisation for coronary heart disease, and region (large cities, Southern, Northern).

As statins vary in how hydrophilic or lipophilic they are, and since lipophilic agents have better brain penetrance, we were interested if agents that were lipophilic or hydrophilic differed. Lipophilic agents when pooled (OR 0.92, 95% CI, 0.88-0.96; p < 0.001) but not hydrophilic agents (OR 0.85, 95% CI 0.70-.103; p = 0.85) demonstrated reduction in risk for depression.

In Table 3 the results of multivariable logistic regression for the association between statin medication and development of depression, adjusted for all independent variables, are presented by increasing age group. A significant stepwise decrease in the odds of depression following statin use, with increasing age was observed (OR ≥ 40 years = 0.95, p = 0.16; OR ≥ 50 years = 0.91, p < 0.001; OR ≥ 60 years = 0.85, p < 0.001; OR ≥ 70 years = 0.81, p < 0.001).

**Table 3:** **Adjusted ORs from multivariable logistic regression for association between statin prescription and subsequent diagnosis of depression between 2006-2008, by increasing age group (** ***n*** **= 4,607,990)**

	**Age ≥40 yrs**			**Age ≥50 yrs**			**Age ≥60 yrs**			**Age ≥70 yrs**		
	**OR** ^**a**^	**95% CI**	**p-value**	**OR** ^**a**^	**95% CI**	**p-value**	**OR** ^**a**^	**95% CI**	**p-value**	**OR** ^**a**^	**95% CI**	**p-value**
**Statin prescriptions**												
Any statin	0.95	0.91-0.99	0.016	0.91	0.87-0.95	<0.001	0.85	0.81-0.89	<0.001	0.81	0.76-0.87	<0.001
Simvastatin	0.93	0.89-0.97	0.001	0.90	0.86-0.94	<0.001	0.85	0.81-0.90	<0.001	0.82	0.76-0.87	<0.001
Pravastatin	0.90	0.74-1.09	0.268	0.84	0.69-1.04	0.104	0.80	0.64-1.01	0.061	0.72	0.54-0.96	0.026
Fluvastatin	0.98	0.65-1.46	0.914	0.91	0.59-1.39	0.660	0.89	0.55-1.43	0.662	1.03	0.60-1.78	0.906
Atorvastatin	1.11	1.01-1.22	0.032	1.04	0.94-1.16	0.427	0.95	0.84-1.07	0.384	0.93	0.80-1.10	0.412
Rosuvastatin	1.21	0.92-1.60	0.177	1.18	0.88-1.60	0.274	1.10	0.75-1.60	0.635	1.21	0.71-2.04	0.485

CI = confidence interval; OR = odds ratio.

^**a**^The model includes prescription of statin medication (any statin, and by individual statins) and the following variables as covariates: gender, age (40-49, 50-59, 60-69, 70+), marital status, education (three levels), family income (quartiles), hospitalisation for cardio-pulmonary disease, hospitalisation for alcoholism, hospitalisation for coronary heart disease, and region (large cities, Southern, Northern).

## Discussion

The results of this study suggest that use of any statin medication is associated with a reduced risk of subsequent diagnosis of depression, decreasing the risk of developing depression by as much as 5% after adjusting for possible confounders. Of the individual statins, simvastatin, by far the most widely used agent, was associated with a 7% reduction in the risk of developing depression, whereas atorvastatin was associated with an 11% increase in the risk of developing depression. Associations were not significant for other individual statins, likely due to the small numbers of depression cases found amongst the less commonly prescribed statins. Furthermore, lipophilic agents but not hydrophilic agents were associated with a reduction in risk of depression. There was a stepwise decrease in risk of depression following statin use with increasing age group, with the highest adjusted decrease in risk (approximately 20%) for those aged 70 years and over.

These data are consistent with findings from a number of previous smaller studies which have examined the relationship between statin use and depression in both population based and clinical samples, although to our knowledge this is the largest study to date and the only one to have utilised a national cohort. Feng et al. [25], examined the relationship between statin use and depressive symptoms in a population-based sample of 2804 community-dwelling adults aged 55 years and over. They found a significant negative association between statin use and depressive symptoms (OR = 0.71, 95% CI 0.52-0.97). Depressive symptoms in this study were determined using a depression rating scale designed specifically for older people, as opposed to register data. Similarly, Pasco et al. [9] in a study of 386 population based women aged over 50 years, found women taking either statins or aspirin for at least 6 months were less likely to develop de novo major depression as determined by a clinical interview, with an age-adjusted OR for major depressive disorder of 0.15 (95% CI 0.03-0.65) in the case-control component of the study, and a hazard ratio of 0.2 (95% CI 0.04-0.85) for the retrospective cohort analysis. In a prospective cohort study involving 965 outpatients with coronary heart disease, Otte et al. [27] found statin use to be associated with both a 34% decrease in risk of depressive symptoms at baseline compared to non-statin use (OR = 0.66, 95% CI 0.45-0.98), and a 33% decrease in risk over the 6 year follow-up period (OR = 0.67, 95% CI 0.48-0.92).

In a prospective follow-up study of 193 patients hospitalised for a cardiac event, Stafford and Berk [24] utilised data from clinical interviews and self-report rating scales to examine the relationship between post-discharge statin use and later development of depression. They found a 69% decrease in the risk of developing depression 3 month post discharge for those prescribed any statin medication compared to no statin medication (OR = 0.31, 95% CI 0.097-0.972), and a 79% decrease in risk after 9 months (OR = 0.21 95% CI 0.052-0.876). In another study utilising a clinical sample, Young-Xu et al. [17] found that among patients with coronary artery disease (n = 606), long-term statin use (4-7 years) was associated with a reduction in the risk of increased depression scores (OR = 0.63, 95% CI 0.43-0.93). Longer length of exposure was associated with a progressive reduction in levels of depression, with the impact only being seen after a minimum of one year of statin treatment. This association with duration of statin use was also found by Yang et al. [26]. Their analysis of data from a case-control study of 94,441 primary health patients found a significantly lower risk of first-episode depression for patients exposed to statin therapy (OR = 0.4, 95% CI 0.2-0.9) compared with those receiving either non-statin lipid lowering therapy or no lipid lowering therapy. The association was stronger for those who were exposed to statin therapy for a year or more, as compared to those with less than a year of exposure.

Contradictory findings have, however, been reported in other studies. In a study of 1,070,706 patients discharged on either statin therapy or no statin therapy from Swedish hospitals following ischemic stroke, Asplund et al. [23] found no difference in self-reported low mood or prescription of antidepressants after 3 months. The inclusion in this study of only ischemic stroke patients, the short follow up period and the broader age range (18 years and over) may account for the findings. Feng et al. [29], in a prospective observational study of 1,803 participants aged 55 and over found there was no difference in depressive symptoms between users and non-users of statins after 1.5 years. Post-hoc analysis revealed statin use may be associated with a reduction in symptoms for women and an increase in symptoms for men, however the evidence for this was not conclusive.

A number of randomised control trials have compared the effects of individual statins, including lovastatin [20,30], pravastatin [21,30], and simvastatin [18,19,22] on psychological wellbeing, mood and/or depressive symptoms versus placebo. None of these studies found statin use to be protective against depression or negative changes in wellbeing or mood, with Hyyppa et al. [18] finding that use of simvastatin was associated with an increase in depression after 24 weeks in hypercholesterolemic men (n = 120). Similarly, Morales et al. [19] reported use of simvastatin and a decrease in self-reported positive affect in elderly volunteers with raised serum cholesterol after 15 weeks (n = 80). The relatively short follow-up periods and small number of depressed patients in both these studies, coupled with differences in the characteristics of the samples compared to the current study, limit their generalisability and are likely to influence the differences in results.

### Strengths of the study

Undoubtedly the greatest strength of this study was the large sample size, which increases the validity of the results. The size of the cohort also allowed for individual statins to be investigated separately, which a number of previous studies have been unable to do due to smaller sample sizes or as a result of an experimental study design [31]. This is important as there have been some suggestions that certain statins have more or less of an effect on mood than others, potentially as a result of their hydrophilic or lipophilic properties [31]. While some of the less commonly prescribed statins in our study did not have sufficient identified depression diagnoses to provide the required power to find significant associations, we were able to show a significant negative association between use of simvastatin, the most widely used agent, and subsequent development of depression, and a significant positive association between use of atorvastatin and subsequent development of depression. Given the smaller numbers of individuals on atorvastatin, the power of the analysis was less than that for simvastatin. As hypothesised, benefits were seen for pooled lipophilic but not hydrophilic statins. As both simvastatin and atorvastatin are classified as lipophilic statins, lipophilicity does not appear a plausible explanation for their divergent effects.

Furthermore, the use of a national cohort reduces the possibility of selection bias and increases the generalisability of these results, as it comprises a representative and diverse group of individuals across a range of socio-economic, ethnic, language, education and regional backgrounds. Consequently these results may be generalisable beyond Sweden to similarly aged citizens of other comparable high-income countries, particularly the other Nordic countries. Use of national patient and pharmacy records to obtain data could also be regarded as a strength of the study, eliminating the possibility of recall bias which can be a problem in studies utilising self-report questionnaires to obtain data on past medication use and depressive symptoms.

### Limitations of the study

The main methodological limitation of this study, as with any observation study, was the potential undermining of internal validity due to unknown and unequally distributed confounding factors across the groups being compared. Many well-known potential confounders were adjusted for, although it is possible that other unidentified confounders (such as medications, physical co-morbidities or lifestyle factors) have had an impact on the results. Body Mass Index (BMI) in particular is known to be associated with both depression and cardio-vascular conditions leading to statin use, however data on BMI was not able to be obtained using the available data sources for this study. History of hospitalisation for COPD and alcohol dependence were used as proxies for smoking and alcohol use in an attempt to control for lifestyle factors known to be associated with depression and coronary heart disease, as such information is not readily accessible through inpatient or outpatient registers. While adjusting for such risk factors is important to decrease the likelihood of confounding, these are imperfect proxies and these indicators were only able to identify a small minority of the total number of alcohol users and smokers within the cohort, being those with the most severe health consequences of their behaviour. Lack of information on serum cholesterol level is also a limitation of the study. It should be noted that while this study, conducted in a Swedish population cannot necessarily be extrapolated to other populations [28], these results are concordant with other published studies. Statins have clear vascular effects, and as such may impact the risk for vascular depression, a particular factor in the elderly, and as such their effects may not fully support the inflammatory hypothesis [32]. Antidepressant effects in post-stroke depression are concordant with this potential [33].

Individuals with a previous diagnosis of depression and other psychiatric disorders were not excluded from this study, unlike some previous studies [9,19-21]. The exclusion of these individuals may have decreased the likelihood of confounding impacting on the results, however the high prevalence of mental disorders in the community would have resulted in the exclusion of a large proportion of the cohort and furthermore would have limited the generalisability of the findings [31].

The use of clinical diagnoses and prescription of antidepressant medication from patient registers to identify cases of depression rather than clinical interviews or questionnaires may have resulted in an underestimation of the true number of depression diagnoses during the study period, especially amongst those not utilising health care services on a regular basis (who would be less likely to be identified and assessed for possible depression), or those being treated solely with psychological and other non-biological treatments. Due to the increased likelihood that those individuals prescribed statins during the study period were seen with some regularity by a health care practitioner, it is possible that this could constitute a differential information bias with the underestimation being more prominent amongst those not on statin therapy. If this were the case we could expect our results to be an underestimate of the true association, pulling the results away from the null hypothesis. It’s also possible that these results would pull towards the null hypothesis.

Other limitations of the study were the lack of information on statin dosage, adherence to treatment, and duration of statin use. We have assumed that all those collecting a prescription for a statin medication have in fact taken the medication as prescribed, although it has been estimated that 12-month adherence rates to statins are around 60%, with the risk of non-adherence reducing with increasing age [14,27]. This is a potential source of non-differential information bias within the study due to the likely overestimation in numbers exposed to statin use amongst both those with and without a diagnosis of depression; the likely effect is to under-estimate the strength of the associations. Other studies have found an association between increasing duration of statin use and decreased risk of depression [17], and being unable to control for that in this study may have impacted on the results. Our findings suggesting increasing protection against the risk of depression by increasing age group may be a reflection of longer average duration of statin use in older patients and a higher percentage of use in that population increasing power, however this cannot be confirmed. It is possible that the impact of statins would be different in younger cohorts.

This study cannot provide direct proof of the inflammatory hypothesis of depression. At best, it is indirectly supportive, given that statins have clear anti-inflammatory properties, and that these results are broadly concordant with the published literature showing a potential anti-depressant effect of statin therapy. Definitive proof will come from clinical trials of statins that are able to show that the benefit of statins on mood are mediated by changes in inflammatory biomarkers.

## Conclusions

Our findings support the hypothesis that treatment with statin medications reduces the risk of subsequent development of depression. While the association for any statin use was statistically significant, the strength of the association for those aged 40 years and over was not strong and therefore may not be clinically significant when considering the implications of this finding. Many of the methodological limitations of the study such as reliance on clinical diagnosis and assumptions regarding adherence risk biasing the study towards underestimation of effects. This issue is further complicated by the fact that our findings showed differences in the effects of individual statins, with simvastatin appearing to have a protective effect on the development of subsequent depression and atorvastatin appearing to increase the risk of depression. These results may go some way to explaining why previous studies have failed to conclusively confirm or refute the hypothesis that statin use may have an effect on mood and depressive symptoms, as the vast majority have either examined the effects of only one or two individual statins, or conversely have included many statins but only analysed the combined effect of the group. Further research comparing the effects of individual statins on depression seems warranted, although this would require the inclusion of sufficient numbers in order to ensure conclusive and accurate results.

If, as has been hypothesised previously, some statins are indeed able to provide protection against depression through their action on oxidative and inflammatory processes [9,10], this may guide the development of novel biological treatments and preventive strategies beyond conventional antidepressant medications, which may have overt public health significance. Further research to confirm the strength of these protective effects and to determine which statins are most effective is needed.
